# Hierarchical Requirement for Endothelial Cell Connexins Cx37, Cx47, Cx43, and Cx45 in Lymphatic Valve Function

**DOI:** 10.1093/function/zqaf034

**Published:** 2025-07-26

**Authors:** Michael J Davis, Jorge A Castorena-Gonzalez, Min Li, Alexander M Simon, R Sathish Srinivasan

**Affiliations:** Department of Medical Pharmacology & Physiology, University of Missouri, Columbia, MO 65212, USA; Department of Pharmacology, Tulane University School of Medicine, New Orleans, LA 70112, USA; Department of Medical Pharmacology & Physiology, University of Missouri, Columbia, MO 65212, USA; Department of Physiology, University of Arizona School of Medicine, Tucson, AZ 85724, USA; Cardiovascular Biology Research Program, Oklahoma Medical Research Foundation, Oklahoma City, OK 73104, USA

**Keywords:** GJC1, GJC2, GJA4, GJA1, lymphedema

## Abstract

The proper functioning of lymphatic valves is critical for unidirectional lymph transport. Valve development and maintenance depends on multiple genes in lymphatic endothelium, including those controlling the expression of 4 connexin (Cx) isoforms—Cx37, Cx47, Cx43, and Cx45. The relative importance of these isoforms for valve function is undefined, but primary human lymphedema is linked to loss-of-function mutations in Cx47 or Cx43, while deficiencies in Cx43 or Cx45 produce functional valve defects in mice. Tests of back leak and closure for single lymphatic valves from mice with selective deficiency of each Cx isoform revealed defects associated with the loss of Cx37 or Cx43, but not Cx47. Combined deletion of multiple isoforms, including Cx45 but not Cx47, produced even more severe valve defects in certain genotypes, sometimes with nearly complete regression of valves within 6 d. Back leak across connexin-deficient LVs correlated highly with gaps between the commissures formed by leaflet insertion into the vessel wall, indicating that connexin function may be critical for the formation and/or maintenance of leaflet commissures. Our results reveal the following hierarchy of Cx importance in valve function: Cx37 = Cx43 > Cx45 > Cx47 and predict that patients with loss of function mutations in Cx37 (*GJA4*) should develop lymphedema. We propose a general classification scheme describing 4 stages of progressive valve dysfunction.

## Graphical Abstract

**Graphical Abstract:**
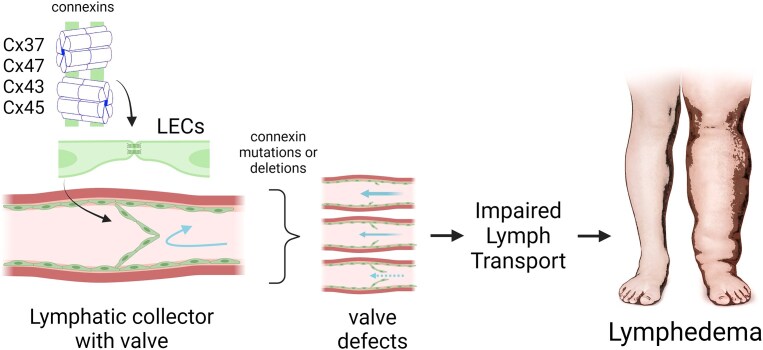

## Significance Statement

The function of lymphatic valves is in part determined by the expression of 4 connexin (Cx) isoforms—Cx37, Cx47, Cx43, and Cx45—whose relative importance is undefined. Functional tests of single lymphatic valves from mice with selective deficiency of each Cx isoform revealed defects associated with the loss of Cx37 or Cx43, but not Cx47. Defects in Cx-deficient LVs correlated with gaps between leaflet commissures and even complete valve regression when certain Cx isoforms were deleted in combination, suggesting that Cx function may be critical for commissure formation. Our results reveal a hierarchy of Cx importance for valve function: Cx37 = Cx43 > Cx45 > Cx47 and predict that patients with loss of function mutations in Cx37 (*GJA4*) should develop lymphedema.

## Introduction

A major function of the lymphatic system is to reabsorb excess fluid and protein from the interstitium for return to the central veins. Reabsorption occurs over the large surface area for water and solute exchange created by extensive networks of lymphatic capillaries in almost every tissue. Lymphatic capillaries drain into precollectors and collectors, each containing intraluminal lymphatic valves (LVs) that prevent back flow and are therefore critical for ensuring unidirectional lymph movement. The buildup of excess interstitial fluid and protein as a result of lymphatic dysfunction leads to the chronic disease lymphedema.

Hereditary forms of lymphedema occur as a result of genetic mutations in a number of critical genes that govern the development of lymphatic vessels and/or valves.^1-3^Connexins are one class of proteins with inherited mutations linked to human lymphedema. During LV development connexin (Cx)-mediated communication between lymphatic endothelial cells (LECs) is presumably important for the coordinated migration, condensation and elongation of LECs to form specialized valve leaflets.^4^ Multiple studies associate Cx mutations with both primary and secondary human lymphedema,^5-10^ but the underlying mechanisms are not fully understood.

Mouse models are increasing used to study the mechanisms underlying lymphedema. Four Cx isoforms are expressed in mouse lymphatic endothelium: Cx37, Cx47, Cx43, and Cx45, encoded by *Gja4, Gjc2, Gja1*, and *Gjc1*, respectively.^11-16^ Deficiencies in each isoform are associated with different aspects of altered LV numbers and/or morphology. Defects in LV function have been documented in mice deficient in Cx45^11^ and to a lesser extent, Cx43,^17,18^ but quantitative information on valve function is lacking for Cx37 and Cx47. *Cx37*^−/−^ mice lack venous valves, exhibit reduced densities of diaphragmatic and dermal LVs^19,20^ and show tracer backflow in some lymphatic networks,^4,21^ suggestive of valve incompetence, but measurements of actual LV function in *Cx37*^−/−^ mice are lacking. Likewise, venous valves are absent in virtually all peripheral veins of *Cx47*^−/−^ mice, but those mice exhibit normal numbers of LVs and normal expression of canonical LV markers without reflux of injected tracer,^19,22^ which is perplexing because patients with Cx47 missense mutations develop lymphedema;^6,7,9^ measurements of LV function in *Cx47*^−/−^ mice are also needed.

The goal of the present study was to systematically evaluate the importance of Cx37, Cx47, and Cx43 in LV function by combining genetic mouse models of Cx isoform deficiency with quantitative physiological tests of LV function. The results show that loss of either Cx37 or Cx43 is particularly critical for normal LV function whereas loss of Cx47 is not. Comparisons of LVs deficient in each of the 4 LEC Cx isoforms revealed a hierarchy for Cx requirement in valve function: Cx37 = Cx43 > Cx45 > Cx47. Based on our combined measurements of back leak and valve closure, we propose a new classification system to describe the progression of valve defects associated with connexin deficiency that likely applies to the loss of other critical proteins required for valve development and/or maintenance.

## Methods

### Ethical Approval

All animal procedures were approved by the animal care committee at the University of Missouri (protocol #9797) and complied with the standards stated in the “Guide for the Care and Use of Laboratory Animals” (National Institutes of Health, revised 2011).

### Mice

*Cx47^−/−^, Cx37^−/^* ^−^, *Cx43^+/−^, Cx43^fl/fl^*, and *Lyve1-Cre; Cx43^fl/fl^* mouse lines have been described previously.^4,18,21,23-26^  *Cx45^fl/fl^* mice were a gift from Klaus Willecke, University of Bonn, Bonn, GDR. *Prox1CreER^T2^* mice were a gift from Taija Mäkinen, Uppsala University, Uppsala, Sweden.

*Cx37^−/−^* and *Cx43^+/-^* mice were interbred to generate *Cx37^+/−^; Cx43^+/−^* mice. *Cx37^−/−^* and *Cx43^±^* lines were bred to *Cx47^−/*−*^* mice to generate *Cx37^−/−^; Cx47^−/−^* and *Cx43^+/-^; Cx47^−/*−*^* mice, respectively. To delete Cx45 embryonically, we bred *Lyve1-Cre* mice with *Cx45^fl/fl^* mice to obtain *Lyve1-Cre; Cx45^fl/fl^* mice. Likewise, embryonic deletion of Cx43 was accomplished by breeding *Lyve1-Cre* mice with *Cx43^fl/fl^* mice to obtain *Lyve1-Cre; Cx43^fl/fl^* mice. Mice used for valve test studies carried germ line deletions of one Cx45 or Cx43 allele and were designated as *Lyve1-Cre; Cx45^Δ/lf^* or *Lyve1-Cre; Cx43^Δ/lf^* mice, respectively. The Lyve1-Cre will also delete Cx45 or Cx43 from macrophages, so we cannot rule out a possible role of macrophage-mediated Cx expression in valve development, even though there is no existing evidence for such an effect. The *Prox1-CreER^T2^; Cx43^fl/fl^; Cx37^−/−^* line was generated in house by crossing *Cx37^−/−^, Cx43^fl/fl^*, and *Prox1-CreER^T2^* mice. *Cx45 ^fl/fl^*mice were bred to *Prox1-CreER^T2^; Cx43^fl/fl^; Cx37^−/−^* mice to obtain *Prox1-CreER^T2^; Cx43^fl/fl^; Cx45 ^fl/fl^; Cx37^−/−^* mice. *Lyve1-Cre; Cx43^fl/fl^* mice were bred to *Cx45^fl/fl^* mice to generate *Lyve1-Cre Cx43^fl/fl^; Cx45^fl/fl^* mice. However, only 5 such mice were obtained over a 3-yr period and one died by 4 wk. The other 4 mice, which were used for valve test studies between 5 and 6 wk, carried a germ line deletion of one Cx43 allele and thus were designated as *Lyve1-Cre Cx43^Δ/fl^; Cx45^fl/fl^* mice. All mouse lines were based on the *C57Bl/6* background strain, “WT,” or were backcrossed into that strain for at least 6 generations. All genotypes of mice used in these studies were comprised of both sexes.

For genotyping, genomic DNA was extracted from tail clips using the HotSHOT method. Genotypes were determined by PCR with 2xM-PCR OPTI Mix (Catalog #B45012, Bimake, Houston, TX, USA) according to the provider’s instructions. For postnatal deletion, *Prox1-CreER^T2^* mice containing one or more floxed Cx genes were induced with 5 consecutive daily i.p. injections of 100 mg tamoxifen (10 mg/mL; safflower oil), after P30. Mice were studied at 2-6 mo after birth for valve function tests.

### Vessel Isolation and Cannulation

Mice were anesthetized with ketamine/xylazine (100/10 mg/kg, i.p.) and placed in the prone position on a heated tissue dissection/isolation pad. Popliteal lymphatic vessels were exposed through a superficial incision in the leg, removed and transferred to a Sylgard-coated dissection chamber filled with Krebs-albumin solution for further dissection. Mesenteric collectors were isolated by opening the abdomen, removing the entire small intestine and pinning it onto a Sylgard-coated 60-mm tissue culture dish. Individual popliteal or mesenteric (duodenum) collectors were pinned to the dissection chamber using 40 μm wire. After removing the majority of the associated fat and connective tissue, vessels containing 3-4 valves were then transferred to a 3-mL myograph chamber containing Krebs-albumin solution and cannulated at each end with a glass micropipette (40-50 μm OD tip), pressurized slightly and further cleaned. The chamber, with attached micropipettes, pipette holders and micromanipulators was transferred to the stage of an inverted microscope. Polyethylene tubing connected the back of each micropipette to low pressure transducers and a computerized pressure controller^11^ allowing independent control of inflow (Pin) and outflow (Pout) pressures. Pin and Pout were briefly set to 10 cmH_2_O at the beginning of every experiment and the vessel segment was stretched axially to remove longitudinal slack. After the pressures were returned to 3 cmH_2_O, the vessel was allowed to equilibrate in Ca^2+^-free Krebs buffer at 37°C for 20-30 min to eliminate spontaneous contractions (in the case of popliteal vessels) that otherwise would have interfered with valve function tests. Constant exchange of buffer at a rate of 0.5 mL/min was maintained using a peristaltic pump. Custom LabVIEW programs (National Instruments; Austin, TX, USA) acquired analog data from the pressure transducers at 30 fps simultaneously with vessel inner diameter, which was detected from video images acquired using a Basler firewire camera^27^ Digital videos of the valve function protocols, with embedded pressure data, were recorded for additional off-line analyses if needed.

### Valve Function Tests

After a multi-valve segment was mounted on the microscope, the segment length was measured and the total number of valves counted. The segment was then shortened to a single valve for valve function tests and the rest of the vessel was stored at room temperature for later re-cannulation and study. A 10-μm initial hole was made with a sharply tapered pilot micropipette, which was then removed and replaced with a more gradually tapered servo-null micropipette (tip diameter = 3-5 μm) to measure luminal pressure on the inflow side of the valve (Psn). After insertion, the servo-null micropipette was advanced to seal the hole. The calibration of the servo-null pipette was adjusted as needed after raising Pin and Pout simultaneously between 0.5 and 10 cmH_2_O. Not all valves could be studied: some were spaced too close together to allow cannulation and subsequent insertion of a servo-null pipette.

To ensure accurate and consistent measurements of valve back leak, (1) all 3 transducers (Pin, Psn, Pout) were calibrated before each experiment; (2) the same pair of cannulation pipettes was used for all experiments to maintain consistent resistances; (3) the pipettes were cleaned after each experiment with boiling water and acetone and checked before each valve test to ensure that the tips were free of debris and, if needed, debris was cleared by sliding fine suture several times through the tip); (4) the pipettes and cannulation tubing were free of bubbles; (5) the Psn pipette calibration was checked at the beginning and end of each valve test.

The first test of valve function measured pressure back leak through a closed valve. Starting with Pin and Pout = 0.5 cmH_2_O and the valve open, Pout was raised to 10 cmH_2_O, ramp-wise, over a 35-s period while Pin was held at 0.5 cmH_2_O. Normal valves closed as Pout exceeded ∼1 cmH_2_O and remained closed for the duration of the Pout ramp. In some cases where valve leaflets appeared stiffer than normal, gentle tapping of the Pout line was used to encourage closure. Pressure back leak through the closed valve was measured with the servo-null micropipette on the inflow side of the vessel, which could resolve changes as small as ∼0.05 cmH_2_O. The value of Psn at the end of the ramp, minus the value of Pin, was used as a representative index of back leak, but additional values of Psn at intermediate Pout levels were also determined offline using a LabView program by binning the data in 1 cmH_2_O Pout intervals.

The second test determined the adverse pressure gradient (ΔP, Pout—Pin) required to close an initially open valve. As demonstrated previously,^28,29^ this value increased with increasing vessel diameter. The measurements therefore were made over a wide range of baseline pressures, each of which in turn determined the baseline diameter. Starting with the valve open, output pressure was raised ramp-wise and the ΔP was determined at the instant of valve closure. The test was repeated for baseline pressures 0.1, 0.2, 0.3, 0.5, 1, 2, 3, 5, 8, 10 cmH_2_O, resulting in tests over a range of diameters spanning ∼40% to 100% of the maximal passive diameter. ΔP for closure was then plotted against baseline pressure or normalized diameter after the experiment. The highest ΔP that could be tested was 30 cmH_2_O (equating to a maximum Pout of 40 cmH_2_O when Pin was 10 cmH_2_O) without exceeding the specified safe range of the pressure sensor elements. For ease of presentation and statistical analysis, the closure test data at Pin = 0.5 cmH_2_O was used as a representative value of the entire ΔP for closure versus diameter relationship.

### Measurements of Valve Leaflet and Gap Dimensions

Measurements of leaflet dimensions were made for each valve under brightfield illumination. The side views of the valves used in functional tests did not permit the entire leaflet (the curved insertion paths of the leaflets from their common base to their tips) to be visualized, nor was a measurement of the cusp length usually possible. As an alternative, the best approximations of the leaflet lengths, a and a’ (as in ref.^29^ were measured while focusing on the lower surface of the vessel, and then the corresponding approximations of leaflet lengths in the opposite wall (b and b’) were measured while focusing on the upper surface of the vessel. Valve symmetry was defined as the ratio of (a + a’)/2 : (b + b’)/2.

### Solutions and Chemicals

Krebs buffer contained: 146.9 mm NaCl, 4.7 mm KCl, 2 mm CaCl_2_·2H_2_O, 1.2 mm MgSO_4_, 1.2 mm NaH_2_PO_4_·H_2_O, 3 mm NaHCO_3_, 1.5 mm Na-HEPES, and 5 mm d-glucose (pH = 7.4). A buffer of the same composition (“Krebs-BSA”) also contained 0.5% bovine serum albumin. Krebs-BSA buffer was present both luminally and abluminally during cannulation, with the abluminal solution constantly exchanged with Krebs during the experimental protocol. For Ca^2+^-free Krebs, 3 mm EGTA replaced CaCl_2_·2H_2_O. Purified BSA was obtained from (US Biochemicals; Cleveland, OH, USA), MgSO_4_ and Na-HEPES from ThermoFisher Scientific (Pittsburgh, PA, USA) and all other chemicals were obtained from Sigma-Aldrich (St. Louis, MO, USA).

### Statistics

Microsoft Excel was used to compile the initial data and to calculate back leak pressures (Pout-Psn) and values of ΔP for closure (Pout-Pin). Igor (Wavemetrics, Lake Oswego, OR, USA) was used for the display of representative traces. LabVIEW was used to read raw data from text files and bin that data into discrete Pout intervals for subsequent statistical analysis. Prism (v10; Graphpad, San Diego, CA, USA) was used for summary plots and statistical analyses. Specific statistical tests are described in the figure legends, with non-parametric tests used if data sets were not normally distributed. For all figures, data are presented as mean ± SEM and significance is marked as: *P < 0.05; **P < 0.01; ***P < 0.001; ^****^P < 0.0001; unmarked comparisons were not significant at *P *< 0.05. Vessel numbers (*n*) and animal numbers (*N*) are indicated in the figure legends.

## Results

### Valve Back Leak

**Figure 1.**
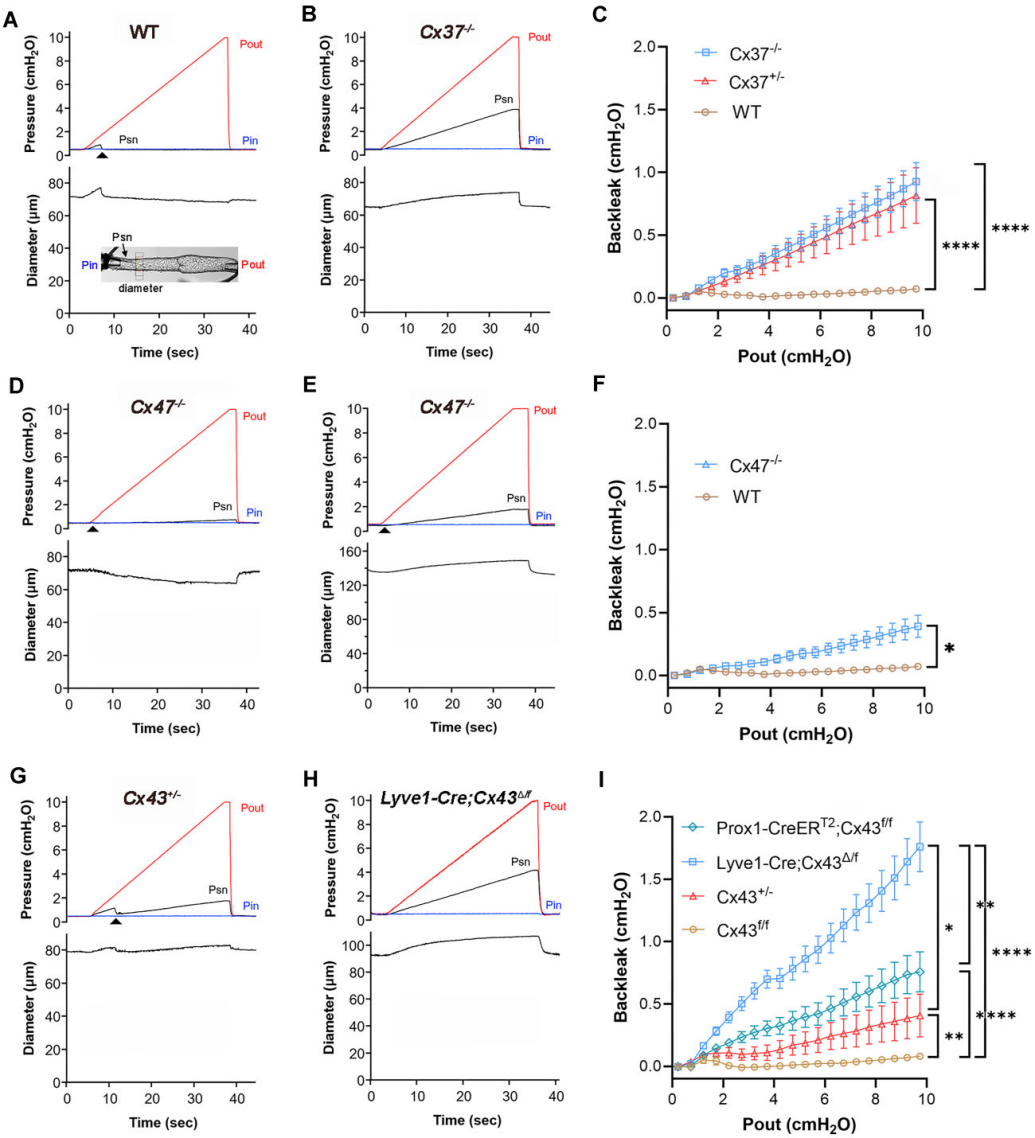
Back leak in WT, Cx37-, Cx47-, and Cx43-deficient LVs. (A) Protocol for determining back leak across a closed LV. Inset shows measurements of Pin, Pout, Psn, and diameter during a protocol conducted on a popliteal lymphatic vessel containing a single valve. With Pin held at 0.5 cmH_2_O, Pout was raised from 0.5 to 10 cmH_2_O. Psn rose until the valve closed (arrowhead) at Pout ∼1.5 cmH_2_O. Back leak at Pout = 10 cmH_2_O (calculated from Psn-Pin) was zero. (B) LV from a *Cx37^−/−^* mouse in which the valve never closed and Psn rose to 3.9 cmH_2_O at Pout = 10 cmH_2_O; back leak = 3.4 cmH_2_O. (C) Back leak tests on 32 WT valves (*N* = 13), 15 *Cx37^+/−^* valves (*N* = 4), and 31 *Cx37^−/−^* valves (*N* = 12), with data binned according to Pout increments of 0.5 cmH_2_O. (D) Test on a *Cx47^*−*/ −^* valve that closed but showed modest back leak (0.3 cmH_2_O). (E) Another *Cx47^−/−^* valve with intermediate back leak (1.3 cmH_2_O). (F) Back leak tests on 32 WT valves (*N* = 13) and 23 *Cx47^−/−^* valves (*N* = 5). (G) Test on a *Cx43^+/−^* valve that closed when Pout reached 2 cmH_2_O, at which point Psn dropped back to 0.5 cmH_2_O, but because the valve did not seal, Psn (and diameter) subsequently rose during the remainder of the Pout ramp; back leak = 1.3 cmH_2_O. (H) Test on a *Lyve1-Cre Cx43^Δ/fl^* valve, which never closed during the Pout ramp; back leak = 3.7 cmH_2_O. (I) Summary data for back leak tests on 10 *Cx43^fl/fl^* control valves (*N* = 6), 14 *Cx43^+/−^* valves (*N* = 3), 24 *Lyve1-Cre; Cx43^Δ/fl^* valves (*N* = 11) and 23 *Prox1-CreER^T2^; Cx43^fl/fl^* valves (*N* = 7), as determined by Kruskal-Wallis tests with Dunn’s post-hoc tests.

A first test of valve function measured pressure back leak across a closed valve as an indicator of how well the valve leaflets sealed under an adverse pressure gradient. The protocol is illustrated in Figure 1A for a WT vessel, with the insert showing an image of the preparation. As Pout was raised to 10 cmH_2_O, Psn began to rise but returned to 0.5 cmH_2_O when the valve closed (arrowhead). After closure, there was no detectable back leak through the valve. In contrast, a *Cx37^−/−^* valve did not close at any time during a similar ramp, with Psn rising to 3.9 cmH_2_O at Pout = 10 cmH_2_O (Figure 1B); net back leak = 3.4 cmH_2_O [calculated from (Psn—Pin) with a theoretical maximum ∼4.75 cmH_2_O]. The average back leak as a function of Pout for 31 LVs from 16 WT mice and 28 LVs from 12 *Cx37^−/*−*^* mice is shown in Figure 1C. The digitized Psn data (minus the respective values of Pin) were binned in 0.5 cmH_2_O intervals according to the Pout level and averaged over each interval. Back leak was also determined for 15 LVs from 5 *Cx37^+/−^* mice (haplodeficient in *Gja4*) and the average data were nearly identical to those of valves from *Cx37^−/−^* mice (Figure 1C). Back leak in both genotypes was significantly elevated in comparison to WT valves, but there were no significant differences between valves from *Cx37^+/−^* and *Cx37^−/*−*^* mice. These results suggest that even haplodeficiency of *Gja4* results in a substantial fraction of LVs with intermediate-to-severe back leak.

Examples of back leak tests for 2 *Cx47^−/−^* valves are shown in Figure 1D-E. The first valve showed barely detectable back leak (0.3 cmH_2_O), whereas the second (Figure 1E) exhibited substantial back leak (1.8 cmH_2_O). Summary data for 23 LVs from 8 *Cx47^−/*−*^* mice are plotted in Figure 1F, compared to the WT data set reproduced from Figure 1C. Average back leak in LVs from *Cx47^−/*−*^* mice was significantly higher than in WT controls, but substantially less than that observed for *Cx37^−/−^* mice. These results suggest that *Gjc2* deficiency results in a significant, but relatively minor, degree of LV back leak.

Representative back leak tests for LVs from a *Cx43^+/−^* mouse and a mouse with constitutive deletion of *Gja1* from LECs are shown in Figure 1G-H. As Pout increased in the test in panel A, the valve closed (arrowhead) but did not seal well, so that Psn rose to an intermediate value of 1.8 cmH_2_O at Pout = 10 cmH_2_O (back leak = 1.3 cmH_2_O). In contrast, the valve from the *Lyve1-Cre; Cx43^Δ/lf^* mouse (panel B) never closed during the Pout ramp and Psn rose to a near-maximal value of 4.2 cmH_2_O. The same pattern is evident in the summary data for 10 valves from 2 *Cx43^+/−^* mice and 20 valves from 10 *Lyve1-Cre; Cx43^Δ/lf^* mice (Figure 1I). Some of the data for *Lyve1-Cre; Cx43^Δ/lf^* mice in Figure 1I are from new tests of popliteal LVs, which were combined with a comparable data set on popliteal LVs of the same genotype published previously^18^ and data from mesenteric LVs of the same genotype from a different published study^17^; these groups are identified in the next figure. New data from *Prox1-Cre; Cx43^f/f^* mice showed an intermediate level of back leak and new data from *Cx43^+/−^* mice indicated modest but significant back leak. The results suggest that haplodeficiency of *Gja1* produces somewhat modest back leak defects in LVs, whereas complete deletion of *Gja1* from LECs (using *Prox1-CreER^T2^* or *Lyve1-Cre*) produces much more severe back leak, with constitutive deletion of *Gja1* (using *Lyve1-Cre*) being associated with the most severe LV dysfunction.

**Figure 2.**
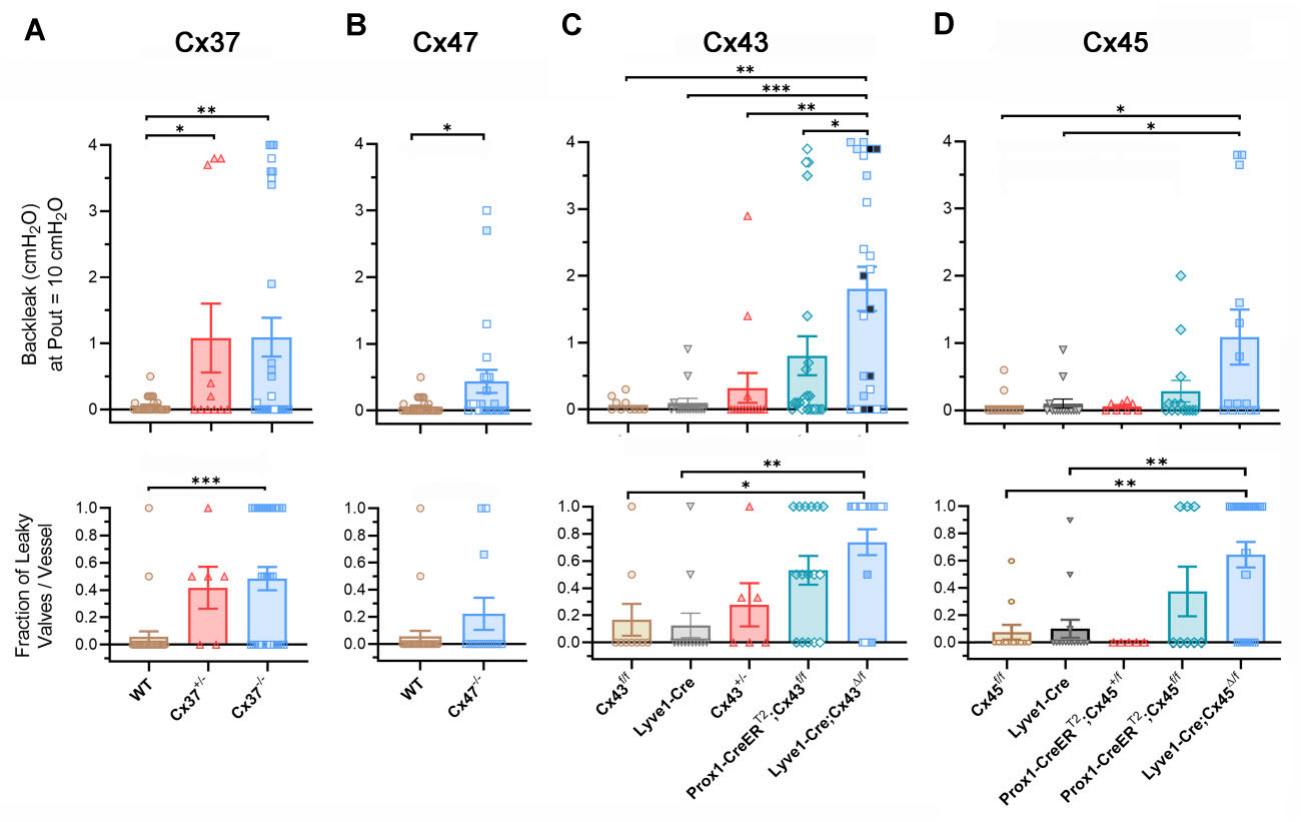
Comparison of back leak in Cx37-, Cx47-, Cx43-, and Cx45-deficient valves. (A-D) In the upper panels, the average back leak at Pout = 10 cmH_2_O is shown for each valve. Popliteal valves are represented by (open) colored symbols and mesenteric valves by white-filled symbols. The white symbols in C are mesenteric valve data replotted from^17^; the black symbols in C are popliteal valve data replotted from^18^; blue symbols in C are new data. The data in panel D replotted from^11^ and shown here for comparison to panels A-C. The lower panels are plots of the fraction of leaky valves for each vessel as a function of genotype. The threshold for a leaky valve (0.11 cmH_2_O) was the mean + one SD for the combined control groups (WT, *Lyve1-Cre, Cx43^fl/fl^*, and *Cx45^fl/fl^*). Comparisons made to the respective “control” genotype using one-way ANOVAs (or *t*-tests in B) with Dunn’s multiple comparison post-hoc tests. *N* and *n* values are stated in Figure 1.

To compare back leak between valves deficient in Cx37, Cx47, Cx43, or Cx45, a single value of back leak at Pout = 10 cmH_2_O for each valve was plotted as a function of genotype (Figure 2A-D). A wide distribution of back leak values in each group (ranging from no back leak to complete valve incompetence) is apparent, accounting for the rather large variance in some of the groups. An alternate method of analyzing the back leak data is shown in the lower panels. For that analysis, the data for each genotype were partitioned into the percentage of vessels with leaky valves, with a leaky valve defined as any valve with back leak greater than the mean + one standard deviation (threshold = 0.11 cmH_2_O) of the combined control groups (WT, *Lyve1-Cre, Cx43^fl/fl^*, and *Cx45^fl/fl^*). For each genotype, the fraction of leaky valves per vessel closely aligned with the average back leak values of the respective genotypes in the top graphs. The summary data suggest the following conclusions: (1) Either complete or haplodeficiency of Cx37 results in significant back leak defects. (2) Cx47 null valves have relatively modest back leak defects. (3) Haplodeficiency of Cx43 does not produce significant back leak, but constitutive deletion of Cx43 from LECs (and macrophages) using *Lyve1-Cre* produces profound back leak defects as does *Prox1CreER^T2^*-mediated Cx43 deletion after the valves have formed. (4) Only constitutive deletion of Cx45 using *Lyve1-Cre* produces significant back leak defects [data are replotted from a recent study^11^ for comparison].

### Valve Closure

**Figure 3.**
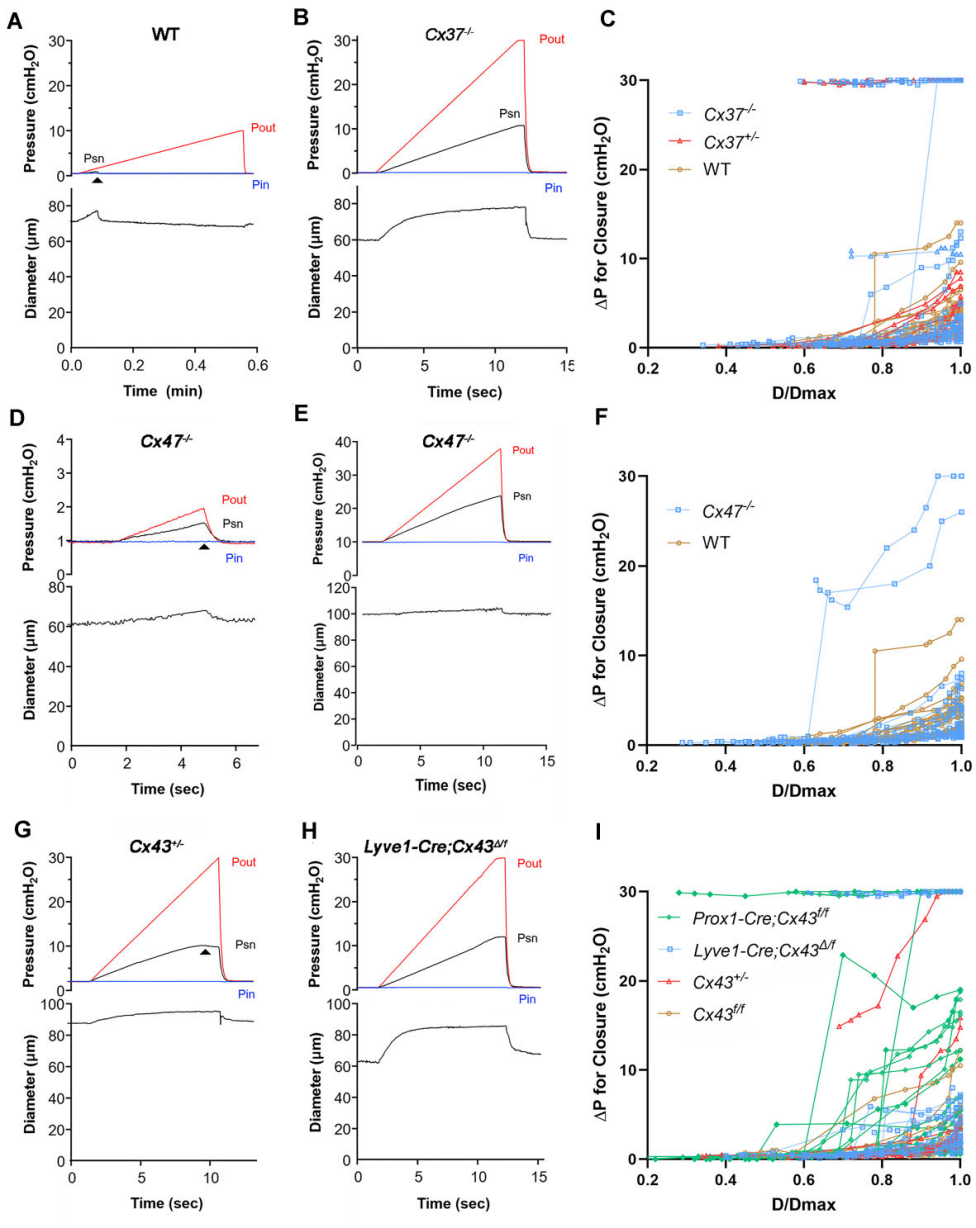
Closure tests for WT, Cx37-, Cx47-, and Cx43-deficient valves. (A) Protocol to determine the adverse ΔP required for closure. With pressures = 1 cmH_2_O, Pout was increased ramp-wise until the valve closed, as indicated by rapid reduction in Psn (arrowhead). At the moment of closure, ΔP (Pout-Pin) = 3.5 cmH_2_O. (B) A similar test on a *Cx37^−/−^* valve in which the valve never closed even at Δ*P* > 29 cmH_2_O. (C) Summary data for closure tests on 27 WT valves, 27 *Cx37^+/−^* valves and 11 *Cx37^−/*−*^* valves with ΔP measured at multiple values of Pin and individual data points plotted against normalized diameter. Normally, the relationship between ΔP and D/Dmax was curvilinear. Incompetent valves are represented by nearly horizontal lines at ΔP ∼30 cmH_2_O. In addition to 3 incompetent *Cx37^+/−^* valves and 5 incompetent *Cx37^−/−^* valves, 5 intermediate valves are evident. One valve required ΔP ∼ 10 cmH_2_O to close even at lowest diameter and another closed normally at low diameters but became incompetent at diameter > 0.8 D/Dmax. (D) A closure test on a *Cx47^−/−^* valve at Pin = 1 cmH_2_O. During the ramp the valve closed when Δ*P* = 1 cmH_2_O. (E) Closure test on the same *Cx47^−/−^* valve at Pin = 10 cmH_2_O, which closed only when Pout reached 38 cmH_2_O (Δ*P* = 28 cmH_2_O). Note different *y*-axis scale. (F) Data for 21 *Cx47^−/−^* valves showing 18 normal valves, one requiring higher than normal ΔP at diameters >0.8 D/Dmax, and two that were partially incompetent. (G) Closure test for a *Cx43^+/−^* valve at Pin = 2 cmH_2_O. The valve partially closed only at Δ*P* = 24 cmH_2_O but did not seal sufficiently to lower Psn. (H) Closure test for a *Lyve1-Cre; Cx43^Δ/fl^* valve at Pin = 0.5 cmH_2_O. The valve never closed even at Δ*P* > 29.5 cmH_2_O. (I) Summary data for 8 *Cx43^fl/fl^* (control) valves, 14 *Cx43^+/−^* valves, 22 *Lyve1-Cre; Cx43^Δ/fl^* valves and 23 *Prox1-CreER^T2^; Cx43^fl/fl^* valves. Three *Lyve1-Cre; Cx43^Δ/fl^* valves and four *Prox1-CreER^T2^; Cx43^fl/fl^* valves were incompetent. Seven of the other 19 *Prox1-CreER^T2^; Cx43^fl/fl^* valves and 2 of 10 *Cx43^+/−^* valves required an abnormal ΔP to close. *N* values are stated in Figure 1.

A second test of valve function measured the adverse pressure gradient required to close an initially open valve, which is thought to reflect leaflet stiffness^28^; the higher the ΔP for closure, the stiffer the valve. The ΔP value for closure could be abnormal even with a normal back leak if the leaflets are unusually difficult to close but seal well once they close. With Pin and Pout set to equal values and starting from a relatively low baseline pressure (0.5 cmH_2_O), Pout was increased ramp-wise until the valve closed (Figure 3A). Closure was evident by a rapid fall in Psn (at arrow). The difference between Pout and Pin at the moment of valve closure was designated as the adverse pressure gradient (ΔP) required for closure. The adverse ΔP was typically <2 cmH_2_O for normal valves at low values of Pin; in the example shown in Figure 3A, Δ*P* < 1 cmH_2_O at Pin = 0.5 cmH_2_O. This value varies up to 20-fold with diameter, as determined by the initial pressure (Pin) and by the passive pressure-diameter relationship for the vessel.^28^ The increase in ΔP with increasing vessel diameter most likely occurs as the leaflets are placed under increasing tension, making closure more difficult—a conclusion supported by numerical modelling of valve structure.^30,31^ An example of an incompetent *Cx37^−/−^* valve, which never closed even when Pout reached 30 cmH_2_O, is given in Figure 3B. The measured values of adverse ΔP for closure at 11 different levels of Pin from 0.1 to 10 cmH_2_O were then used to generate a complete curve describing the relationship between ΔP as a function of normalized diameter (D/Dmax) for each LV. For normal valves this relationship was typically curvilinear, starting at a value ∼0.3 cmH_2_O and rising to ∼2-4 cmH_2_O at D/Dmax = 1. About 10% of WT valves required a slightly higher adverse ΔP for closure, consistent with previous findings.^29,32^ The ΔP versus D/Dmax curves for 22 WT valves are plotted in Figure 3C. Many of the *Cx37^+/−^* and *Cx37^−/−^* valves also showed normal ΔP versus D/Dmax relationships (Figure 3C), but subpopulations of each (*n* = 3 of 11 and 5 of 30, respectively) required an abnormally high adverse ΔP to produce closure or were completely incompetent.

Closure tests for 2 *Cx47^−/−^* valves are shown in Figure 3D-E. The first example shows a relatively normal valve that closed at an adverse Δ*P* = 1 cmH_2_O, starting from a baseline pressure of 1 cmH_2_O (the Pout ramp was terminated immediately after the valve closed). The second example shows the same *Cx47^−/−^* valve, which required a substantially higher than normal ΔP (28 cmH_2_O) to close when starting from a baseline pressure of 10 cmH_2_O. The data sets of closure tests for all the *Cx47^−/−^* valves are shown in Figure 3F, in comparison to the data sets for WT control valves. None of the *Cx47^−/−^* valves were completely incompetent and only 2 of 11 showed abnormal ΔP versus D/Dmax relationships.

Closure tests for Cx43-deficient valves are shown in Figure 3G-H. In panel G, a *Cx43^+/−^* valve did not close until Pout reached 26 cmH_2_O, starting at a baseline pressure of 2 cmH_2_O, and closure (arrowhead) attenuated but did not prevent the subsequent rise in Psn as the Pout ramp continued. This behavior indicates that the leaflets, although closed, did not seal well. Panel H shows a *Lyve1-Cre; Cx43^Δ/lf^* valve that was completely incompetent—never closing even as Pout reached 30 cmH_2_O. Summary data for Cx43-deficient valves, along with data for *Cx43^fl/fl^* control valves, are shown in Figure 3I. Only one *Cx43^+/−^* valve demonstrated a highly abnormal closure test, whereas the closure curves for most *Prox1-CreER^T2^; Cx43^fl/fl^* valves were shifted upward (suggestive of increased leaflet stiffness), and 2 of 9 *Prox1-CreER^T2^; Cx43^fl/fl^* valves were completely incompetent. Complete valve incompetence was also observed in 6 of 22 valves from *Lyve1-Cre; Cx43^Δ/lf^* mice.

**Figure 4.**
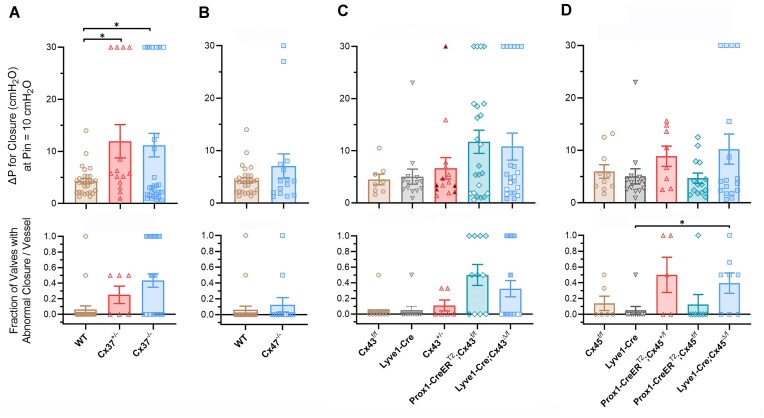
Summary of closure test data for Cx-deficient valves. (A-D) Upper panels: a single value of ΔP for closure at Pin = 10 cmH_2_O is shown for each valve. Popliteal valves are represented by colored symbols, mesenteric valves by filled white symbols. Lower panels: fraction of valves with abnormal ΔP for closure calculated for each genotype. The threshold value for abnormal ΔP was determined from the mean ΔP + one SD for the 4 control groups (WT, *Cx43^fl/fl^, Cx45^fl/fl^*, and *Lyve1-Cre*) = 8.60 cmH_2_O. The data in panel D were previously published^11^ and shown for comparison. Differences were determined using one-way ANOVAs (or *t*-tests in B) with Dunn’s multiple comparison post-hoc tests to the respective “control” genotype. *N* values are stated in Figure 1.

Closure test data for Cx37, Cx47, Cx43, and Cx45-deficient valves are summarized in Figure 4. In general, the results parallel the summary data for back leak in Figure 2, with a few exceptions. Complete or haplodeficiency of Cx37 produced significant defects in the ability of LVs to close under an adverse pressure gradient. *Cx47^−/−^* valves showed no significant closure defects. Haplodeficient Cx43 valves and *Prox1-CreER^T2^; Cx43^fl/fl^* valves exhibited no significant closure defects, and only constitutive deletion of Cx43 or Cx45 resulted in significant closure defects.

### Deletion of Multiple Cx Isoforms

**Figure 5.**
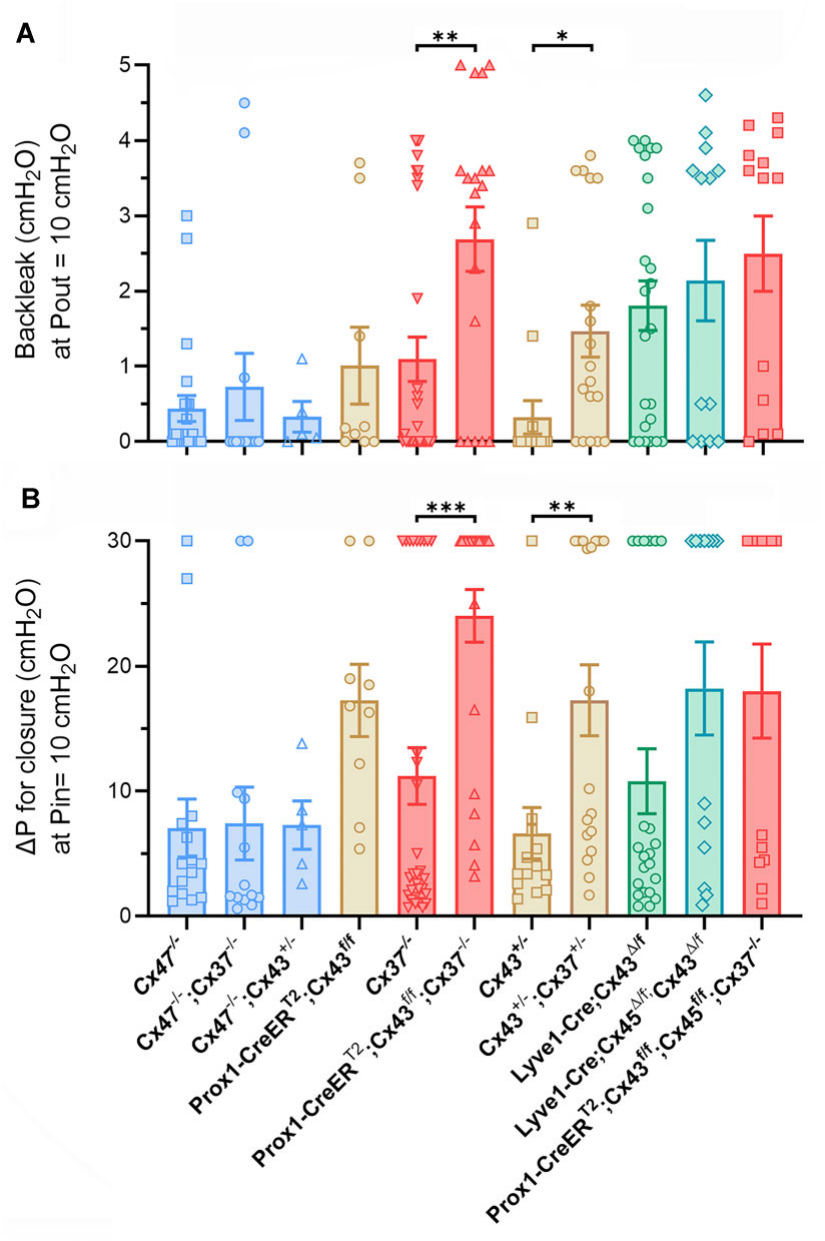
Consequences of combinatorial Cx deletion. (A) Back leak at Pout = 10 cmH_2_O plotted for each genotype. (B) ΔP for closure at Pin = 10 cmH_2_O plotted for each genotype. Blue symbols = Cx47 deficient, gold symbols = Cx43 deficient, red symbols = Cx37 deficient. Significant differences between 3 groups were determined using one-way ANOVAs with Dunn’s multiple comparison post-hoc tests references to the respective single KO genotype. Differences between 2 groups were determined with unpaired *t*-tests. *N* = 5, *n* = 15 for *Cx47^−/−^; N* = 6, *n* = 13 for *Cx47^−/−^; Cx37^−/−^; N* = 7, *n* = 23 for *Prox1-CreER^T2^; Cx43^fl/fl^; N* = 12, *n* = 31 for *Cx37^−/−^; N* = 8, *n* = 24 for *Prox1-CreER^T2^; Cx43^fl/fl^; Cx37^−/−^; N* = 3, *n* = 14 for *Cx43^+/−^; N* = 5, *n* = 18 for *Cx43^+/−^; Cx37^+/−^; N* = 11, *n* = 24 for *Lyve1-Cre; Cx43^Δ/fl^; N* = 3, *n* = 13 for *Lyve1-Cre; Cx45^Δ/fl^; Cx43^Δ/fl^; N* = 6, *n* = 13 for *Prox1-CreER^T2^; Cx43^fl/fl^; Cx45^fl/fl^; Cx37^−/−^*.

We then examined the effects of combinatorial deletion of Cx isoforms. It was impractical to generate and compare all combinations of double and triple Cx isoform deletion because (1) relatively large numbers of manipulated alleles and complicated breeding strategies would have been needed for some genotypes; and (2) in cases in which the genes were located on the same chromosome, we would have needed to rely on rare recombination events for deletion of both genes (eg, in attempts to generate Cx45/Cx47 double KO mice this did not occur during a 3-yr period). We focused on comparing the functional valve defects between single and double KO vessels (Figure 5). Comparisons of back leak between *Cx47^−/−^* (single KO) and *Cx47^−/−^; Cx37^−/−^* valves or *Cx47^−/−^; Cx43^+/−^* valves revealed no significant differences in back leak between these genotypes (Figure 5A). Differences in back leak between Cx43^+/−^ and *Cx47^−/−^; Cx43^+/−^* valves or between *Cx37^−/−^* and *Cx47^−/−^; Cx37^−/^*^−^ valves were also not significant. These results suggest that deletion of Cx47 does not worsen valve defects in the background of deficiency in another Cx isoform. Haplodeficiency of Cx37 increased the severity of back leak in *Cx43^+/−^* valves as did deletion of Cx43 in Cx37 null valves. None of the other comparisons were significant. A similar analysis of closure defects (Figure 5B) yielded significant differences between the same groups. Collectively, the results suggest that loss of Cx37 in the background of the other Cx-isoform deficiencies is the most significant enhancer of valve defects.

### Changes in Leaflet Density and Dimensions

**Figure 6.**
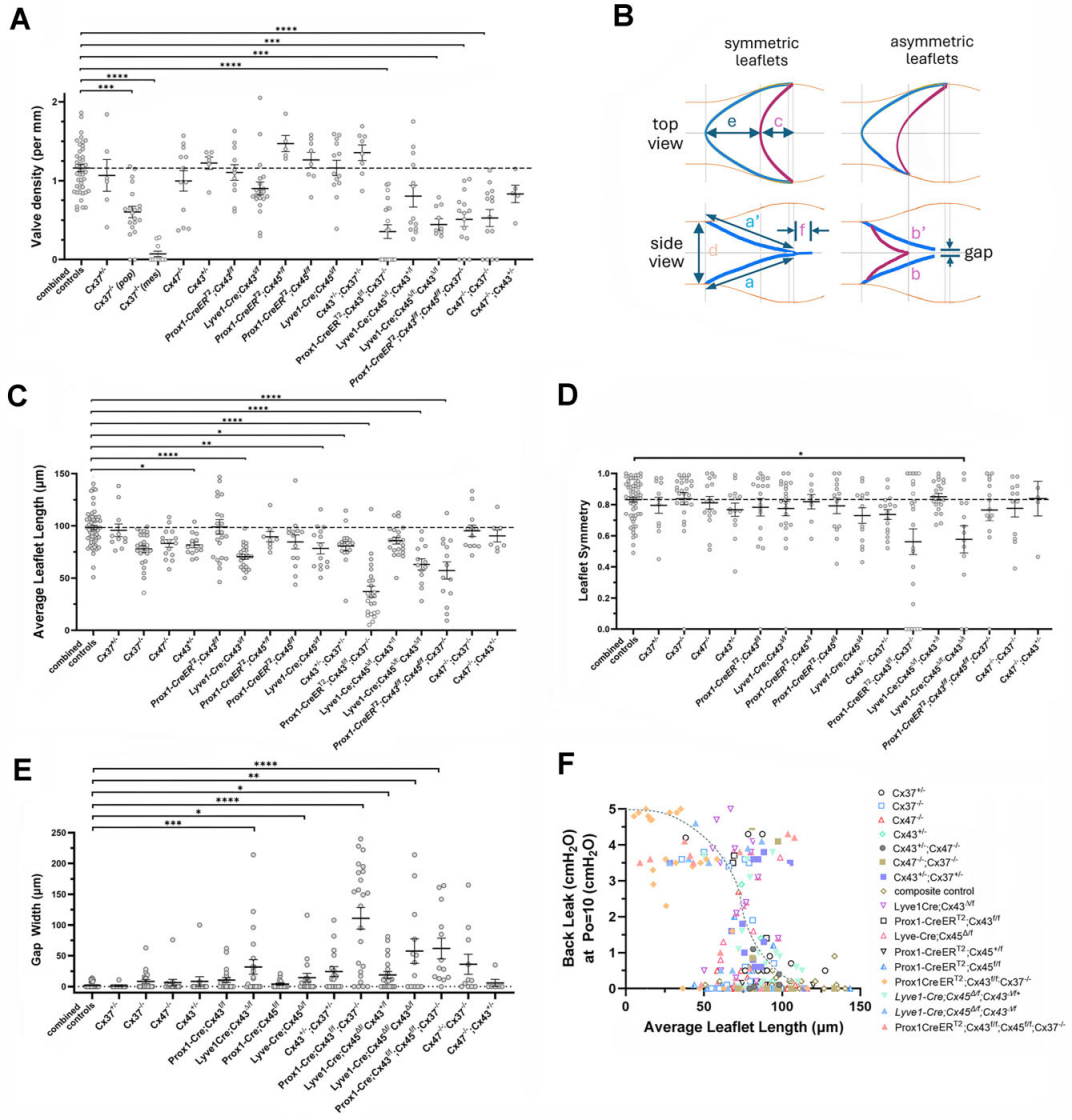
Valve dimension measurements during functional tests. (A) Analysis of valve density. The “combined controls” group is described in text, with dotted line as reference line. (B) Left panel: Schematic of symmetric and asymmetric valves as viewed from the top and side, with dimensions stated previously.^11^ The commissures (with length, “f”) extend beyond the junction of the leaflet tips. Commissure gap is the distance between the ends of the commissures. (C-D) Plots of average leaflet length and leaflet symmetry as stated previously.^11^ (E) Plot of gap width for each genotype. (F) Aggregate plot of back leak versus average leaflet length. Open symbols = single Cx KO valves; Filled symbols = multiple Cx KO valves. Dotted line is an inverse sigmoid function. Kruskal-Wallis non-parametric tests with Dunn’s multiple comparisons post-hoc tests were used to compare valve density, leaflet symmetry and gap width to the combined control group; a one-way ANOVA was used for average leaflet length.

Finally, systematic measurements of valve anatomy were made in the various Cx-deficient mice to gain insight into possible mechanisms whereby Cx-deficiency produces valve dysfunction (Figure 6). Valve densities for the 4 control groups (WT, *Lyve1-Cre, Cx43^fl/fl^, Cx45^fl/fl^*) were not significantly different and thus the data for those groups were pooled as “combined controls” (Figure 6A). Of the 14 different types of Cx-deficient vessels, only 6 showed significant decreases in valve density, with 5 of those involving Cx37 deficiency. The valve density of *Cx37^−/−^* popliteal vessels was only ∼50% of that for the combined controls and the density of *Cx37^−/−^* mesenteric vessels was <10% of that for the combined controls. Valve density was only ∼30% of normal for *Prox1-CreER^T2^; Cx43^fl/fl^; Cx37^−/−^* double KO vessels (popliteal and mesenteric vessels combined), ∼40% of normal for *Prox1-CreER^T2^; Cx43^fl/fl^; Cx45^fl/fl^; Cx37^−/−^* triple KO vessels (popliteal vessels only as no mesenteric valves were present) and ∼30% of normal for *Lyve1-Cre; Cx45^Δ/lf^; Cx43^Δ/lf^* double KO vessels. Further reductions in valve density in *Lyve1-Cre; Cx43^Δ/lf^* and *Prox1-CreER^T2^; Cx43^fl/fl^* mice after the addition of Cx45 deficiency suggest that Cx45 may be compensating for the loss of Cx43.

Under brightfield illumination, we also measured the average lengths of the leaflets and the degree of leaflet asymmetry (see diagrams in Figure 6B). Measurement of true leaflet lengths (the arcs shown in Figure 6B) would have required confocal image stack reconstructions of each valve, which was not feasible without the addition of a fluorescent reporter gene in each mouse line.^11^ The data for average leaflet lengths in control and Cx-deficient valves are summarized in Figure 6C. Most Cx-deficient valves were characterized by shorter leaflets or a single, shorter leaflet, with a lower average length of the 2 leaflets. Leaflet symmetry (Figure 6D) was only significant for *Lyve1-Cre Cx43^Δ/lf^ Cx45^Δ/lf^* valves. Correlations of back leak for each valve against leaflet dimensions allowed us to construct the plot in Figure 6F. An inverse sigmoidal relationship between the degree of back leak and leaflet length [based on the precedent in^18^] is shown, but the deviation of many points from that line suggests that leaflet lengths <100 μm are associated with substantial back leak in ∼50% of LVs and, as leaflet length falls below a minimal value of ∼50 μm, the probability of back leak becomes nearly 100%.

**Figure 7.**
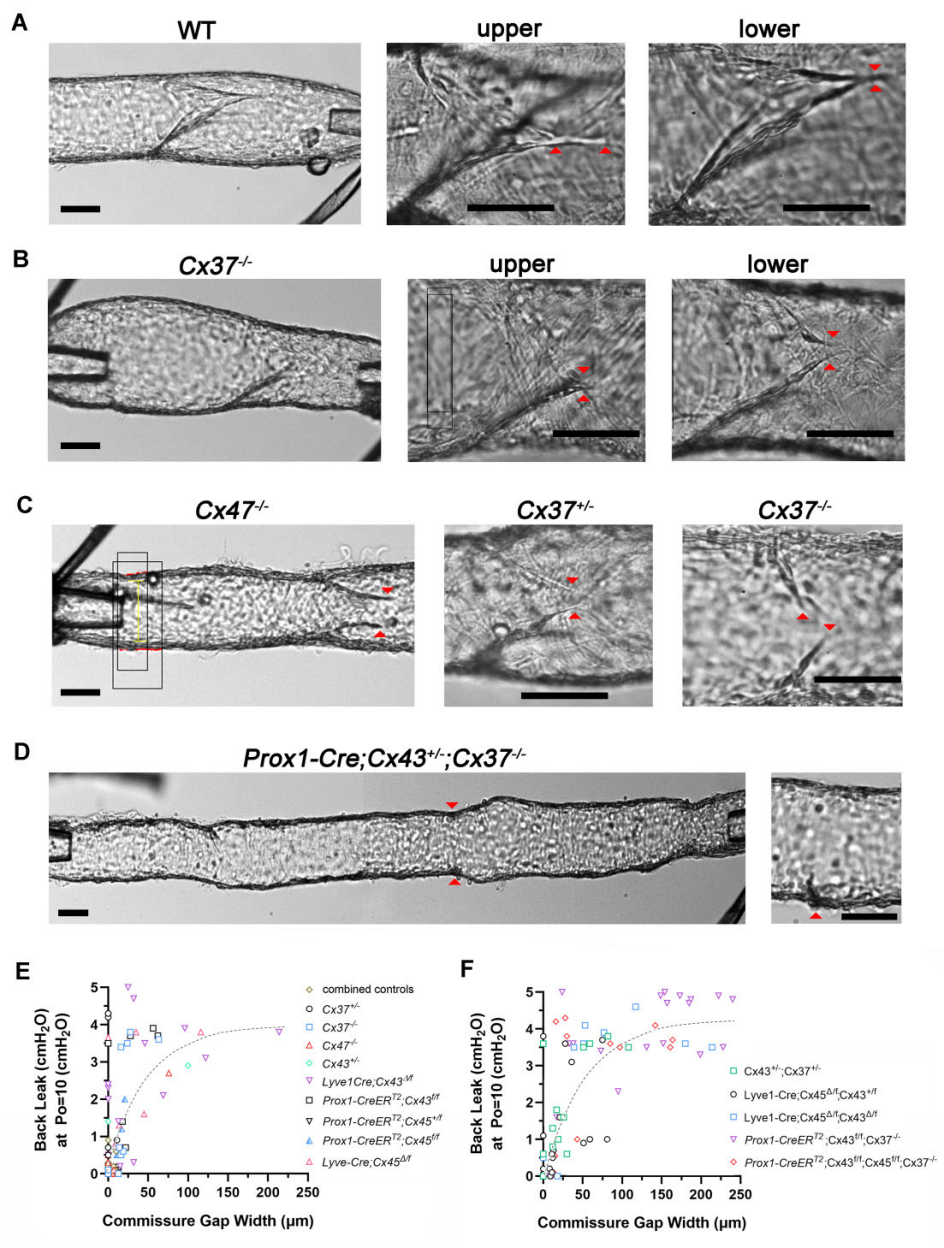
Commissure gaps in control and Cx-deficient lymphatic vessels. (A) WT vessel viewed at 63× (left) and 160× (middle, right). Left arrowhead in the middle panel points to junction of the commissures, the right arrowhead indicates their termination. Arrowheads in the right panel point to the leaflet junction on the other surface of the same vessel. (B) *Cx37^−/−^* vessel at 63× (left) and 160× (middle, right). Arrowheads in middle panel point to the junction of the commissures; arrowheads in right panel show gap between the leaflet tips. (C) Examples of a large gap between leaflets of a *Cx47^−/−^* valve (left), a modest gap between leaflets of a *Cx37^+/−^* valve (middle), and shortened leaflets with large gap in a *Cx37^−/*−*^* valve (right). Black boxes in the left image are diameter tracking windows. (D) Segment of a *Prox1-CreER^T2^; Cx43^fl/fl^; Cx37^−/−^* vessel showing 2 partially regressed valves with varying lengths of leaflets remaining near sinuses. Zoomed image at right shows a single leaflet “nub.” Plots of back leak as a function of the gap between leaflet tips for (E) single Cx-deficient valves and (F) multiple Cx-deficient valves. Curves were fit to equations describing a one-phase association with least squares fit (Prism) *r*^2^ = 0.42 and 0.65, respectively. For panel E, *N* and *n* values are same as in Figures 1 and 3; for panel F, *N* and *n* values are same as in Figure 5. Scale bars are 50 μm.

In a recent study of developing LVs in *Prox1CreER^T2^; Foxo1^fl/fl^; Prox1eGFP* mice, exaggerated gaps between the insertion points of the leaflet tips into the vessel wall were noted in otherwise normal appearing but highly leaky valves.^33^ When analyzing leaflet dimensions in mature LVs in the present study, many Cx-deficient valves also exhibited distinct gaps at this location (see diagram in Figure 6B and summary data for gap width in Figure 6E, which was significantly different from control in several knockout lines). Commissure gaps would seemingly provide a path for back leak to occur at the valve margin even after closure under an adverse pressure gradient. Susceptibility to back leak at this particular anatomical location is also predicted by recent numerical models^30,31^ but has not been tested experimentally. Examples of normal and abnormal commissure gaps are shown in Figure 7. Images of a WT vessel with valve are shown in panel A at 63× and 160× magnification. Under a higher magnification view of the upper surface of the valve, the leaflets can be seen to join at their tips and then extend as a commissure for several tens of microns further into the wall. Commissures were not always present (eg, right panel of Figure 7A), but they were a common feature of most control valves, particularly mesenteric valves. Images of a *Cx37^−/−^* valve are shown in Figure 7B, in which the leaflets joined (and did not extend further) on the upper surface of the vessel, but a distinct gap between the leaflet tips was evident on the lower surface (arrowheads). Likewise, a similar gap was visible on one surface of a *Cx47*-deficient valve (Figure 7C, left panel). Small gaps were sometimes present in control valves, but only 7 of 57 of such vessels exhibited even slight back leak during functional valve tests, and control valves did not have the larger gaps characteristic of many Cx-deficient valves. Commissure gaps were present in a *Cx37^+/−^* valve (middle panel of Figure 7C), whose leaflets were of normal length and closed under an adverse pressure gradient, but the valve exhibited modest back leak (0.4 cmH_2_O). In other cases, such as the *Cx37^−/−^* popliteal valve shown in Figure 7C (right panel), the leaflets were too short to close and overlap under a maximal adverse pressure gradient (back leak = 3.8 cmH_2_O). Extreme cases of shortened leaflets were noted in valves from *Prox1-CreER^T2^; Cx43^fl/fl^; Cx37^*−*/−^* and *Prox1-CreER^T2^; Cx43^fl/fl^; Cx45^fl/fl^; Cx37^−/−^* mice. In both strains, most popliteal valves had regressed within 6 d after post-natal induction, leaving only short nubs at the upstream bases of the valve sinuses (Figure 7D, arrowheads) or unusually long vessel segments (3-4 mm) with no obvious valve leaflets.

A strong correlation between back leak and commissure gap width (taken as the largest gap present on either of the two sides of the valve) was evident, as shown by the plots for all single Cx-deficient genotypes in Figure 7E. Several features of this graph are notable. The steep initial slope of the curve suggested that gap widths <10 μm were not associated with significant back leak; ie, that some minimum gap was required to produce back leak. Gap widths larger than 10 μm were strongly associated with increasing back leak, up to a value of ∼50 μm, at which point back leak was near-maximal. However, the presence of some leaky valves without gaps (ie, points distributed along the *y*-axis origin) suggest that gap width was not the sole determinant of back leak. A similar analysis for valves with deficiencies in multiple Cx isoforms is presented in Figure 7F. Interestingly, although there were many more valves with maximal or near-maximal back leak on this plot, the curve fit for this data set was nearly identical in shape, slope and intercept to that for single Cx-deficient valves, perhaps indicating that once a minimal gap width of ∼50 μm was exceeded, there was a high probability of significant back leak. In summary, the gap width between the commissure tips appears to correlate highly with leaky valves in Cx-deficient mice; however, the substantial scatter in these plots (eg, valves with short leaflets but without back leak) suggests that other factors, such as leaflet stiffness and/or asymmetry, also contribute to back leak.

## Discussion

Our study represents the first quantitative comparisons of LV function in mice deficient in each of the 4 Cx isoforms expressed in mouse LECs. It is also the first assessment of LV function in mice with deletion of *Gjc2* (Cx47), one or both alleles of *Gja4* (Cx37), one allele of *Gja1 (*Cx43), post-natal deletion of Cx43 and deletion of multiple Cx isoforms in combination. Based on the degree of back leak in LVs of the various genotypes shown in Figure 2, we propose the following hierarchy of Cx isoform importance for LV function:


\begin{eqnarray*}
{\mathrm{Cx}}37 = {\mathrm{Cx}}43 > {\mathrm{Cx}}45 > {\mathrm{Cx}}47.
\end{eqnarray*}

An almost identical hierarchy emerges when the degree of back leak and the value of ΔP for closure are combined with the measured structural defects for LVs from the various single and multiple Cx knock mice, as shown in Table 1 (see the rank order in the last column). This order is also consistent with recent scRNAseq analyses of valve LECs that reveal a hierarchy of Cx expression (message): Cx43 > Cx37 > Cx47 > Cx45.^12,16^

**Table 1.** Severity of Valve Functional and Structural Defects After Cx Deletion

Knock out	Score*: 0 (normal) to 5 (severe defect)						Average
	Density	Leaflet Length	Symmetry	Gap Width	Back Leak	ΔP for Closure	
**Cx37**	5	2	0	1	4	4	2.7
**Cx43**	1	3	0	1	5	3	2.2
**Cx45**	0	1	2^#^	1	3	2	1.5
**Cx47**	1	1	0	1	2	1	1.0
**Cx43;Cx37**	3	5	1	5	5	5	4.0
**Cx43;Cx45**	4	1	2	4	4	4	3.2
**Cx43;Cx45;Cx37**	4	4	0	4	5	5	3.7

*Scoring based on relative severity of defects between genotypes, from data in Figures 2, 4, 5, and 6.

^#^compared to Cx45^f/f^ control (12), but milder if compared to combined controls (Figure 6E).

According to our scheme, Cx37 and Cx43 are ranked as approximately equal in functional importance because Cx43 (*Gja1*)-deficient valves had more severe average back leak and the highest percentage of valves with back leak (Figure 2C, 2F), but *Gja4* (Cx37) haplodeficiency produced more severe back leak and a higher fraction of valves with complete incompetence than *Gja1* (Cx43) haplodeficiency (Figure 1C versus Figure 1G). In addition, the densities of LVs in both mesenteric and popliteal networks of *Cx37^−/−^* mice were significantly reduced, while the densities in Cx43-deficient mice were not (Figure 6C). Under a gravitational load, a lower valve density would result in increased continuity of the hydrostatic pressure column that could potentially develop in diastole of the lymphatic contraction cycle, leading to higher diastolic luminal pressure and thereby promoting back leak across any remaining dysfunctional valves. A more accurate assessment of potential systemic consequences for lymph transport would include the relative changes in valve density among the various Cx-deficient genotypes, but such a calculation would be complicated by the fact that changes in LV density appear to be region-specific (Figure 6A).

Based on the combined assessment of back leak and the adverse ΔP for closure, we propose 4 stages of deteriorating valve function that result from Cx deficiency:

1. Slightly abnormal—valves that close at a normal adverse ΔP but exhibit modest back leak;
2. Abnormal—valves that close at a normal adverse ΔP but exhibit substantial back leak;
3. Partially incompetent—valves that close at low adverse ΔP but open at higher imposed adverse ΔP and then become incompetent, with a subsequent high degree of back leak;
4. Completely incompetent—valves with near-maximal back leak that do not close at any adverse ΔP.

These stages are referenced to normal valves (stage 0), which close at an adverse Δ*P* < 2 cmH_2_O and exhibit no detectable back leak. Deletion of any of the 4 Cx isoforms in LECs leads to a variable mixture of these stages for any given genotype, with Cx37- or Cx43-deficient valves having the most severe phenotype (stages 3-4), Cx45-deficient valves having intermediate dysfunction (stages 2-3), and Cx47-deficient LVs having the mildest phenotype (stage 1). Combined deletion of multiple Cx isoforms results in a higher percentage of LVs with stage 4 dysfunction than the deletion of any single Cx isoform, with the simultaneous deletion of Cx47 having little or no additional effect.

### Normal Valves

A consistent and somewhat surprising finding from the present study is that ∼10% of “normal” valves have a slight degree of back leak (stage 1). The reason remains unclear, but it was also noted for other “control” LVs in our previous studies.^29,32,34^ A slight compromise in the function of normal valves is consistent with the elevated turnover rate of LECs in valve regions under relatively high shear stress—about 10% of the LEC population^35^—and may reflect compromised LV function while critical LECs are being replaced. It is also curious that some normal LVs are always preserved in genotypes with combinatorial deletion of multiple Cx isoforms—even genotypes with a high percentage of stage 4 dysfunctional valves (Figure 5). Explanations include the possibilities that some valves are resistant to Cx deficiency, that Cx turnover in certain valve LECs is a very slow process, and/or that deficiency of one isoform can be compensated by other isoforms.

### *Gja4* (Cx37) Deficiency

Our study provides the first quantitative information on the consequences of Cx37 deficiency for LV function. Both Cx37 and Cx47 are required for venous valve formation^19,20^ and Cx37 is reported to be essential for initial assembly of the ring-like valve territory in developing LVs.^4^ In vivo dye backflow studies suggest that LVs in some regions of *Cx37^−/*−*^* mice are leaky,^21^  ^,^^4^ although those tests can be misleading,^11^ but the data in Figures 2A and 4A demonstrate statistically significant back leak defects in Cx37-deficient LVs. Interestingly, in the current study, *Cx37^+/−^* valves displayed defects in function comparable to those of *Cx37^−/−^* valves, suggesting that 2 alleles of *Gja4* are required for normal LV function. Normal numbers of LVs were previously reported in *Cx37^+/-^* mice, but with a reduction in the fraction of fully mature valves assessed by immunostaining.^21,4^  *Cx37^−/−^* mice had reduced densities of mesenteric, dermal, diaphragmatic, and thoracic duct LVs during embryogenesis and in adulthood.^21,4^ Here, we found that LVs were nearly absent in the mesentery lymphatic network of adult *Cx37^−/−^* mice but were only somewhat reduced in density in popliteal lymphatics (Figure 6A). A tentative conclusion is that Cx37 is *not* absolutely required for the formation of all LVs, since they are preserved in some regions of *Cx37^−/−^* mice albeit at a lower-than-normal density. As Cx37 has been proposed to be a critical step in the transduction of OSS leading to LV formation^4^ it is surprising that 50-60% of valves in *Cx37^−/*−*^* mice have *normal* function (Figure 2A), and that mice with combinatorial deletion of Cx43/Cx37, Cx45/Cx43/Cx37, or Cx47/Cx37 still retained *some* normal valves (Figure 5). Possibly another, unidentified, Cx isoform can substitute for the role of Cx37 under these conditions. An implication of our finding that Cx37 haplodeficiency results in dysfunctional valves is that human patients with loss-of-function mutations in one allele of *GJA4* are predicted to experience some degree of primary lymphedema and, indeed, whole exome sequencing of patients with primary lymphedema has recently revealed potential pathological variants of *GJA4* (personal communications from Miikka Vikkula, de Duve Institute, Brussels, Belgium; and Pia Ostergaard, St. George’s University of London). A possibly related observation is that 2 single nucleotide polymorphisms in the 3’UTRs of *GJA4*, which could affect the expression of Cx37 protein, has been documented in breast cancer patients with secondary lymphedema following surgery^36^

### *Gjc2* (Cx47) Deficiency

A surprising finding of our study is that valve defects in Cx47-deficient mice are relatively mild compared to those with deficiencies in any of the other 3 Cx isoforms (Figures 2B, 4B), despite the relatively strong connection between human lymphedema and the (presumed) loss of Cx47 function in patients with *GJC2* missense mutations.^6,7,9^ No decrease in mesenteric LV density was noted in *Cx47^−/−^* mice,^19,22^ and we detected a slight but non-significant decrease in popliteal LV density in those mice (Figure 6C); however, a systematic investigation of valve density in other regional lymphatic networks of *Cx47^−/−^* mice would be needed to make wider conclusions. Cx47 loss-of-function mutations could directly affect LV function, but *Cx47^−/−^* mice do not develop the severe chylothorax observed in *Cx37^−/−^; Cx43^+/−^* mice or in mice with LEC-specific knockout of Cx43.^21^ The data in Figures 2 and 4 suggest that complete loss of Cx47 results in only relatively mild LV back leak with little change in the ability to close. However, lymphedema could potentially develop in patients with *GJC2* mutations if dysfunctional venous valves produce venous insufficiency that results in excess filtration from blood capillaries and overwhelms the transport capacity of the lymphatic system. Compensatory upregulation of other Cx isoforms could occur in *Cx47*^−/−^ mice, but this possibility has not been tested. Alternatively, specific Cx47 mutations might negatively affect the expression or function of other proteins critical for lymphatic function, including other Cx isoforms that potentially interact with Cx47.^18^ In support of this possibility, a missense mutation in the extracellular domains of Cx47, which is associated with dominantly inherited human primary lymphedema, resulted in a 2-fold increase in the number of jugular lymph nodes in mice, and lymph reflux in mesenteric collectors^37^ suggestive of changes in both lymphangiogenesis and valve function. Such missense Cx47 mutations could potentially act as assembly-mediated dominant negative inhibitors if they interfere not only with Cx47 function but also with the activity of a co-expressed lymphatic Cx.

### *Gja1* (Cx43) Deficiency

Our data suggest comparable importance of Cx43 and Cx37 for normal LV function in mice. *Cx43^−/−^* mice do not survive after birth^38^ due to pulmonary edema and obstruction of the right ventricular outflow tract. Haplodeficiency of *Gja1* alone does not reduce the number of mesenteric LVs, but *Cx43^+/−^; Cx37^−/−^* mice exhibit significantly fewer LVs in the thoracic duct than *Cx37^−/−^* mice^21^ Lymphatic-specific deletion of Cx43 using *Lyve1-Cre*, resulted in > 90% decrease in mesenteric LV density at E18.5^18^ In *Cx43^+/−^; Cx37^+/−^* mice, we examined LV density only in the popliteal network, where it was slightly *higher* than control. Our measurements of back leak, combined with those of two previous studies, show that lymphatic-specific deletion of Cx43 results in severely compromised LV function in which 60-75% of LVs are nearly or completely incompetent (Figure 2C). Closure tests for Cx43-deficient valves, not measured previously, show a similar pattern, with 30-50% of LVs requiring an abnormally high ΔP to close (Figure 4C). Our new measurements indicate that 2 alleles of Cx43 are needed for normal valve development because *Cx43^+/−^* mice exhibit LV function that is compromised to almost the same degree as LVs with complete deletion of Cx43 from LECs (Figure 2C). Finally, based on new assessment of LV function in *Prox1-CreER^T2^; Cx43^fl/fl^* mice (Figures 1I, 2C), our results indicate that Cx43 is also required for LV maintenance.

### *GjaC1* (Cx45) Deficiency

We recently reported that constitutive deletion of Cx45, using *Lyve1-Cre*, results in back leak defects and closure defects in a substantial percentage of LVs, whereas deletion of Cx45 after LVs had developed, using *Prox1-CreER^T2^*, resulted in only a few defective LVs^11^ Some of those results were reproduced here for comparison to the other Cx isoforms. New results in this study indicate that Cx45 deletion exacerbated functional defects and reductions in LV density in mice deficient in Cx37 and/or Cx43 (Figures 5, 6), possibly indicating that Cx45 can compensate for loss of Cx37 or Cx43. However, unlike Cx43- or Cx37-deficiency, Cx45 deficiency alone did not alter LV density^11^ In comparison to the other Cx isoforms, the present data suggest that Cx45 is less important for overall LV function than Cx43 or Cx37 but more important than Cx47 (Figures 2B, 4B).

## Conclusions

Our findings lead to the following conclusions: (1) Cx37, Cx43 and Cx45 are each required for the development of competent, non-leaky LVs; (2) Cx43 is the most important isoform for valve maintenance; (3) Cx37 or Cx43 haplodeficiency is sufficient to severely compromise LV function; (4) loss of Cx37 has the greatest impact on LV density; and (5) constitutive Cx47 deficiency only modestly compromises LV function. The data suggest the following hierarchy of Cx isoform importance in LV function: Cx37 = Cx43 > Cx45 > Cx47. Our results predict that patients with loss-of-function mutations in one allele of *GJA4* (Cx37) should develop lymphedema. The association between lymphedema and *GJC2* (Cx47) mutations in humans is puzzling and can tentatively be attributed to a reduced number of LVs in those patients and/or to venous valve insufficiency. The degree of back leak defects in Cx-deficient valves correlates strongly with an increasing gap width between leaflet commissures, but the roles of connexins in this process and the underlying developmental mechanisms remain to be investigated.

Finally, we propose a classification scheme describing 4 stages of progressive LV dysfunction associated with Cx isoform deficiency, ranging from slightly abnormal to completely incompetent (see above). This scheme combines measurements of closure, related to leaflet stiffness, with measurements of back leak, related to the degree of overlap of closed leaflets. The scheme likely applies to loss of other transcription factors or proteins required for LV development and function. For example, a retrospective analysis of data from previous studies suggests that vessels with LEC-specific deletion of *S1pr1* have 50-80% of valves with stage 4 dysfunction^39^ vessels with LEC-specific deletion of *Rasa1, EphB4*, or *Foxc2* have >50% of valves with stage 3-4 dysfunction,^29,32,40^ vessels with LEC-specific deletion of *Tie1* have ∼25% of valves with stage 4 dysfunction^41^  *Foxc2* haplodeficient valves have only stage 1 dysfunction,^17^  ^,^^34^ and mature *Foxo1*-deficient valves have no dysfunction (stage 0).

## Data Availability

All data needed to evaluate the conclusions in the paper are present in the paper. Data will be made available from the corresponding author upon request.
